# Research status and evolutionary trends of digital sports: a perspective of co-word analysis

**DOI:** 10.3389/fspor.2026.1785033

**Published:** 2026-03-19

**Authors:** An Mengbin, Li Mengchen, Ru Xueyan, Jia Junjie

**Affiliations:** 1 Department of Combat Sports and Special Training, Belarusian State University of Physical Culture, Minsk, Belarus; 2 College of Physical Education, Henan Normal University, Xinxiang, China; 3 Graduate Student Department, Jilin Sport University, Changchun, China

**Keywords:** bibliometric analysis, co-word analysis, digital sports, research trend, social network analysis, word frequency analysis

## Abstract

**Introduction:**

Despite the extensive penetration of digital technologies into the field of sports, a comprehensive review of the knowledge structure and developmental trends in this domain remains lacking. This study aims to employ co-word analysis to systematically map, for the first time, the knowledge landscape of international digital sports research from 2005 to 2025, identifying its core themes and evolutionary trends.

**Methods:**

Drawing on a sample of 1,318 core English-language papers from the Web of Science database, this study utilized BICOMB 2.0, SPSS 26.0, and UCINET 6.0 to conduct high-frequency word analysis, social network analysis, and cluster analysis.

**Result:**

A total of 39 high-frequency keywords were extracted, and four main research clusters were identified: Digital Innovation Promoting the Development of Sports Media; Implementation of Digital Devices in Sports Training; Integration of Digital Technology in Physical Education; Impact of Digital Transformation on Sports Health. The study found that “exercise,” “gamification,” and “physical activity” were the most closely interconnected terms, while keywords such as “kinesiology” and “biomechanics” remained at the periphery of the research network.

**Conclusion:**

This study reveals, for the first time, the four major clusters within digital sports research and their underlying socio-technical logics—platformization, the datafication of the body, technological mediation, and the health paradox. It also highlights the disconnect between traditional sports science and digital research. This macro-level framework provides a foundation for subsequent theoretical integration and interdisciplinary dialogue.

## 1 Introduction

Digital technology has irreversibly permeated all aspects of sports, from performance analysis in competitive sports to daily participation in public fitness, from the production and dissemination of sports media to the business models of the sports industry, giving rise to an interdisciplinary field known as “digital sports.” This transformation is not merely the application of digital technology but a profound change involving the reshaping of social relations, cultural practices, and economic structures. Academia has responded rapidly, with a large number of studies emerging from different disciplines such as sports science, computer science, communication studies, sociology, and management, forming a fragmented yet interconnected knowledge map. Despite numerous empirical studies, there is a lack of comprehensive reviews that depict the overall knowledge structure, development trends, and research hotspots of this field from a macro perspective. Existing reviews are mostly confined to specific areas, such as esports, digitalization of sports media, or wearable devices. Therefore, this study adopts literature analysis methods, especially co-word analysis, to objectively reveal the thematic connections and research trends implicit in the literature, providing a macro perspective for understanding the full picture of digital sports development.

However, as a rapidly evolving interdisciplinary field, the concept of “digital sports” itself exhibits a certain degree of ambiguity and inclusiveness. It overlaps with yet differs in emphasis from adjacent fields such as esports, digital health, and sports data analytics. To clarify the scope of this study, this paper operationally defines “digital sports” as: the theoretical and practical domain that utilizes digital information technologies (e.g., big data, sensors, artificial intelligence, virtual/augmented reality, social media platforms, etc.) to empower, reshape, or innovate participation in sports activities, performance enhancement, teaching and training, management and operations, dissemination and consumption, as well as industrial forms (1, 2). Through bibliometric methods, the research aims to reveal the intellectual structure within this broad field, rather than focusing on a specific subset of it (such as purely technical performance analysis or culturally oriented esports research).

From the perspective of bibliometrics, this study systematically delineates the knowledge structure and evolutionary trajectory of digital sports research through co-word analysis and the mapping of scientific knowledge. This approach is rooted in the theoretical frameworks of “research fronts” and “intellectual bases” within bibliometric theory (46); (3). It reveals the latent thematic structure and dynamic evolution of the disciplinary field by analyzing the co-occurrence relationships of keywords in the literature. This study aims to use Bicomb 2.0, SPASS 26.0, and UCINET 6.0 software, through co-word analysis and social network analysis, to achieve the following objectives: (1) Identify high-frequency keywords and core research themes in international digital sports research from 2005 to 2025; (2) Visualize the structural relationships between keywords, identifying research hotspots and development trends within the field; (3) Analyze the evolution trends of research themes and explore the underlying socio-technical drivers. By mapping the scientific knowledge graph of digital sports research, clarifying the systematic relationships among various elements, and grasping future research trends, this study not only helps scholars quickly locate the core and gaps in the field but also provides a basis for reflecting on how digital technology shapes the social role and cultural significance of contemporary sports.

## 2 Methods

This study employs a descriptive and analytical design: first, it presents high-frequency keywords and thematic clusters through descriptive statistics, offering a macroscopic depiction of the field's knowledge structure. Then, it employs social network analysis and cluster analysis to interpret the structural relationships among keywords and the underlying logic of knowledge organization. Finally, the discussion section explores the sociotechnical transformation implications inherent in digital sports. This study utilizes co-word analysis and social network analysis.

Co-word Analysis. Co-word analysis is a type of content analysis that counts the number of times a set of keywords appear together in the same document, reflecting the closeness of relationships between these keywords, thereby analyzing the research hotspots and ideal landscape of the themes represented by these keywords (4). It is generally believed that the more frequently keywords appear together, the closer their relationship. Based on this, cluster analysis and multidimensional scaling analysis are performed on high-frequency keywords in digital sports to reveal relationships between keywords.

Social Network Analysis. Social network analysis is a method for studying social relationships, revealing social networks and operational patterns by analyzing associations between individuals (5). Researchers have found that relationships between keywords can also be analyzed using social network analysis, displaying the relational properties of keywords through knowledge graphs, thereby revealing research hotspots and development trends in the field.

**Figure 1:**
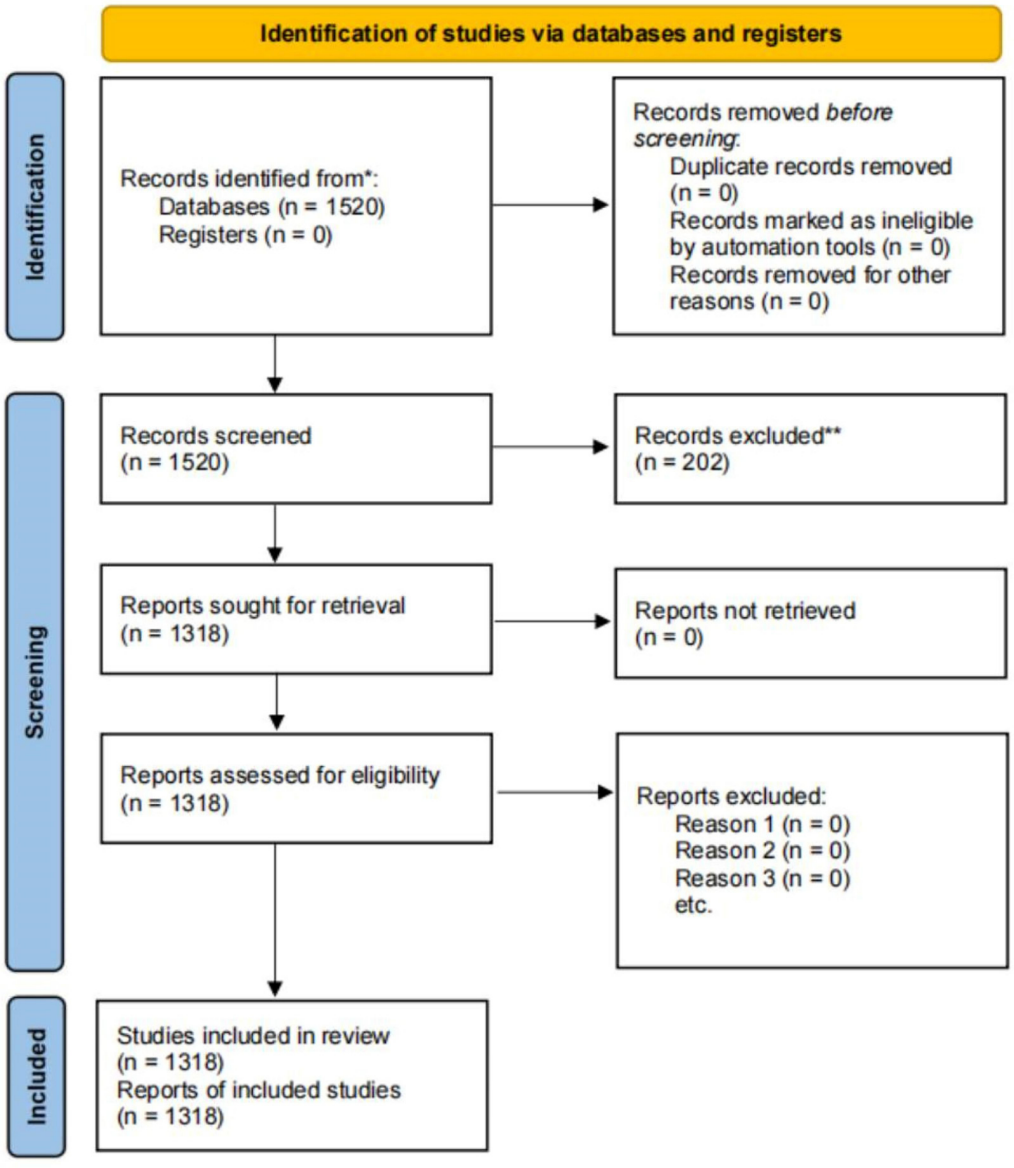
PRISMA 2020 flow diagram.

This systematic review was conducted and reported in accordance with the PRISMA (Preferred Reporting Items for Systematic Reviews and Meta-Analyses) 2020 statement (Figure 1). The study uses Bicomb 2.0 co-word analysis software, UCINET 6.0 social network analysis software, and SPSS 26.0 statistical software as tools. First, the literature is input into Bicomb 2.0 for co-word analysis, where the software performs statistics and analysis on keywords, extracting high-frequency keywords, a word-document matrix, and a co-occurrence matrix. Second, the word-document matrix is imported into UCINET 6.0 social network analysis software to draw a similarity matrix. Finally, SPSS 26.0 statistical software is used to perform cluster analysis and thematic analysis on the similarity matrix.

### 2.1 Data retrieval

Collection of Keyword Samples: This study uses the Web of Science database as the retrieval database, conducting an exact search with the topic: “Digital sports” OR “digital” OR “virtual sports” OR “e-sports,” without specifying journal categories. The retrieval date is July 14, 2025. From the initial retrieval of 1,520 documents, after subsequent screening and removal of 202 documents, a total of 1,318 valid documents were obtained.

This study selects the Web of Science (WoS) Core Collection as the sole data source based on the following considerations: First, WoS is widely recognized as one of the most authoritative academic databases, and its stringent journal selection mechanism ensures the reliability of the literature (6). Second, WoS covers high-impact journals across multiple disciplines, including sports science, communication studies, and computer science, aligning closely with the interdisciplinary nature of digital sports research. Nevertheless, this study also acknowledges the potential limitations of this choice. On one hand, WoS predominantly indexes English-language journals, which may result in the omission of significant research published in other languages, thereby affecting the global representativeness of the findings. On the other hand, WoS has relatively limited coverage of conference proceedings, yet many innovative contributions in digital technology fields are initially presented at top-tier conferences (e.g., ACM, IEEE). The absence of such literature may lead to a lag in capturing research frontiers. Future research could integrate multiple data sources, such as Scopus, PubMed, and Google Scholar, to construct a more comprehensive knowledge map.

### 2.2 Data processing

Extraction of Valid Keywords. Using Bicomb 2.0 co-word analysis software, 1,520 relevant documents were input in txt plain text format, and a total of 7,152 keywords were statistically extracted. The cleaning and merging of keywords followed a semi-automated process: First, Comb 2.0 software was used to automatically calculate the frequency of raw keywords and identify spelling variants (e.g., singular/plural forms, abbreviations). Subsequently, two researchers independently conducted manual verification, merging similar keywords based on a synonym list (e.g., “technologic” and “technology”) and domain knowledge, resulting in 5,726 keywords. Discrepancies were resolved through discussion and consensus. Finally, keywords with broad meanings, such as “sports fandom,” “sports entrepreneurship,” and “sport podcasts,” were removed, yielding 2,670 valid keywords. This method balances efficiency and accuracy, ensuring the standardization of keyword data. Based on the critical value calculation formula for high-frequency and low-frequency words proposed by Sun (7), combined with the distribution of keyword frequencies, the word frequency threshold was finally set to “≥, combin” this threshold ensures that the high-frequency keywords adequately represent the hotspots of the field while avoiding sparse clustering caused by excessively low term frequencies. Calculations identified 39 keywords with a frequency of ≥7, and their cumulative frequency accounts for 32.7% of the total occurrences of all keywords, effectively representing the core research themes in this domain. According to the principle for identifying high-frequency words proposed by Donohue (47), this quantity meets the conventional standards for selecting high-frequency keywords in bibliometric research. The high-frequency keywords are as follows: sport, physical activity, football, physical exercise, Digital health, Digital games, sports journalism, Esport, Digital media, digital, Olympic game, technology, mHealth, Digital technology, gamification, adolescent, injury prevention, health, Internet, Big data, physical education, Wearables, digital journalism, athletes, sensors, Digital transformation, Innovation, game, sports media, reliability, basketball, range of motion, digitalization, mental health, heart rate, sport performance, kinematics, Biomechanics, digital twin. The statistical details of high-frequency keywords are shown in Table 1.

**Table 1:** Statistics of high-frequency keywords.

No.	Keyword	Frequency	No.	Keyword	Frequency	No.	Keyword	Frequency
1	Sport	125	14	Digital technology	15	27	Innovation	11
2	Physical activity	38	15	gamification	15	28	game	10
3	Football	31	16	adolescent	15	29	sports media	9
4	Physical exercise	27	17	injury prevention	14	30	reliability	9
5	Digital health	26	18	health	14	31	basketball	9
6	Digital games	24	19	Internet	13	32	range of motion	9
7	Sports journalism	24	20	Big data	13	33	digitalization	9
8	Esport	23	21	physical education	13	34	mental health	8
9	Digital media	18	22	Wearables	12	35	heart rate	8
10	Digital	17	23	digital journalism	12	36	sport performance	7
11	Olympic game	16	24	athletes	12	37	kinematics	7
12	Technology	16	25	sensors	12	38	Biomechanics	7
13	mHealth	16	26	Digital transformation	11	39	digital twin	7

Construction of Word-Document Matrix and Co-occurrence Matrix. Using Bicomb 2.0 co-word analysis software, the 39 high-frequency keywords were analyzed to obtain a word-document matrix (details in Table 2) and a co-occurrence matrix (details in Table 3). Table 2 shows part of the co-occurrence matrix; the first column and first row are high-frequency keywords. The data can reflect the connection between two keywords; the more frequent the co-occurrence, the closer the connection. It can also correct the accuracy of subsequent analysis and provide intuitive data interpretation. Table 3 shows part of the word-document matrix; the first column lists high-frequency keywords, and the first row corresponds to article numbers (only articles corresponding to high-frequency keywords, not all keywords). “0” indicates that the keyword did not appear in the corresponding numbered article, while “1” indicates that it did appear.

**Table 2:** Co-occurrence matrix (partial).

Keyword	Sport	Physical activity	Football	Physical exercise	Digital health	Digital games
Sport	125	3	6	3	0	3
Physical activity	3	38	0	5	6	2
Football	6	0	31	0	0	1
Physical exercise	3	5	0	27	2	1
Digital health	0	6	0	2	26	1
Digital games	3	2	1	1	1	24
….	….	….	….	….	….	….
Digital health	1	1	1	0	0	2
Kinematics	0	0	0	1	2	1
Sport performance	1	0	0	1	0	0
Digital twin	0	0	0	1	0	0
Physical exercise	1	0	1	0	0	0
Biomechanics	1	0	0	0	0	0

**Table 3:** Word-Document matrix (partial).

Keyword	2	4	5	6	….	1,498	1,510	1,516	1,518
Sport	0	0	1	1	…	0	0	0	0
Physical activity	0	0	0	0	…	0	1	0	0
Football	0	0	0	0	…	0	0	0	0
Physical exercise	0	0	0	0	…	0	0	0	1
Digital health	0	0	0	0	…	0	1	0	0
Digital games	0	0	0	0	…	0	0	0	0
Sports journalism	0	0	0	0	…	0	0	0	0
Esport	0	0	0	0	…	0	0	0	0
Digital media	0	0	0	0	…	0	0	0	0
Digital	0	0	1	0	…	0	0	0	0

Construction of Similarity Matrix. This study employs the Chi-squared (Chiai) coefficient to construct a keyword similarity matrix. Compared to the cosine coefficient or the Jaccard coefficient, the Chi-squared coefficient is less sensitive to low-frequency terms in co-word analysis, allowing for a more robust reflection of the true association strength among high-frequency keywords (48), which aligns well with the analytical objectives of this study. First, the word-document matrix was imported into SPSS 26.0 software in EXCEL format, the Chiai coefficient was selected, and a similarity matrix was generated (details in Table 4). In the similarity matrix, a correlation coefficient value closer to 1 indicates greater similarity between two keywords.

**Table 4:** Similarity matrix (partial).

Keyword	Sport	Physical activity	Football	Physical exercise	Digital health	Digital games
Sport	1.000	0.044	0.097	0.052	0.000	0.059
Physical activity	0.044	1.000	0.000	0.156	0.195	0.071
Football	0.097	0.000	1.000	0.000	0.000	0.039
Physical exercise	0.052	0.156	0.000	1.000	0.077	0.042
Digital health	0.000	0.195	0.000	0.077	1.000	0.044
Digital games	0.059	0.071	0.039	0.042	0.044	1.000
….	….	….	….	….	….	….
Digital health	0.032	0.057	0.064	0.000	0.000	0.154
Kinematics	0.000	0.000	0.000	0.068	0.141	0.077
Sport performance	0.034	0.000	0.000	0.073	0.000	0.000
Digital twin	0.000	0.000	0.000	0.073	0.000	0.000
Physical exercise	0.034	0.000	0.068	0.000	0.000	0.000
Biomechanics	0.034	0.000	0.000	0.000	0.000	0.000

## 3 Results

### 3.1 High-frequency keyword analysis

#### 3.1.1 Centrality

Centrality indicates the centrality of a point and is one of the important indicators in social network analysis. Centrality includes closeness centrality, betweenness centrality, and degree centrality. By calculating and arranging the centrality of high-frequency keywords in digital sports, research hotspots in this field can be effectively explored. This study uses closeness centrality and betweenness centrality to draw the co-word network graph of high-frequency keywords in digital sports. Betweenness centrality is calculated from the binary matrix, and the calculation results are shown in Tables 5, 6. Closeness centrality, also known as global centrality, represents the sum of the shortest paths from one node to other nodes. The smaller the distance of the node, the higher its closeness centrality.

**Table 5:** Top 10 nodes by betweenness centrality in the high-frequency keyword Co-occurrence network.

No.	Betweenness	Centrality
1	Sport	271
2	Physical exercise	50
3	Gamification	42
4	Football	30
5	Digital health	28
6	Big data	27
7	Olympic game	26
8	Physical activity	25
9	Digital games	23
10	Digital	22

**Table 6:** Top 10 nodes by closeness centrality in the high-frequency keyword Co-occurrence network.

No.	Closeness	Centrality
1	Sport	85
2	Gamification	97
3	Physical activity	100
4	Football	101
5	Physical exercise	101
6	Health	102
7	Digital games	102
8	Digital	102
9	Technology	103
10	Big data	105

Thus, it can be seen that the keywords sport, physical exercise, gamification, physical activity, football, Digital games, digital, and Big data occupy important positions in this research field and have a relatively high degree of control. The betweenness centrality of the keywords range of motion, injury prevention, kinematics, and Biomechanics is 0, indicating that these keywords are on the periphery of the research network and are less important.

#### 3.1.2 Social network analysis

**Figure 2:**
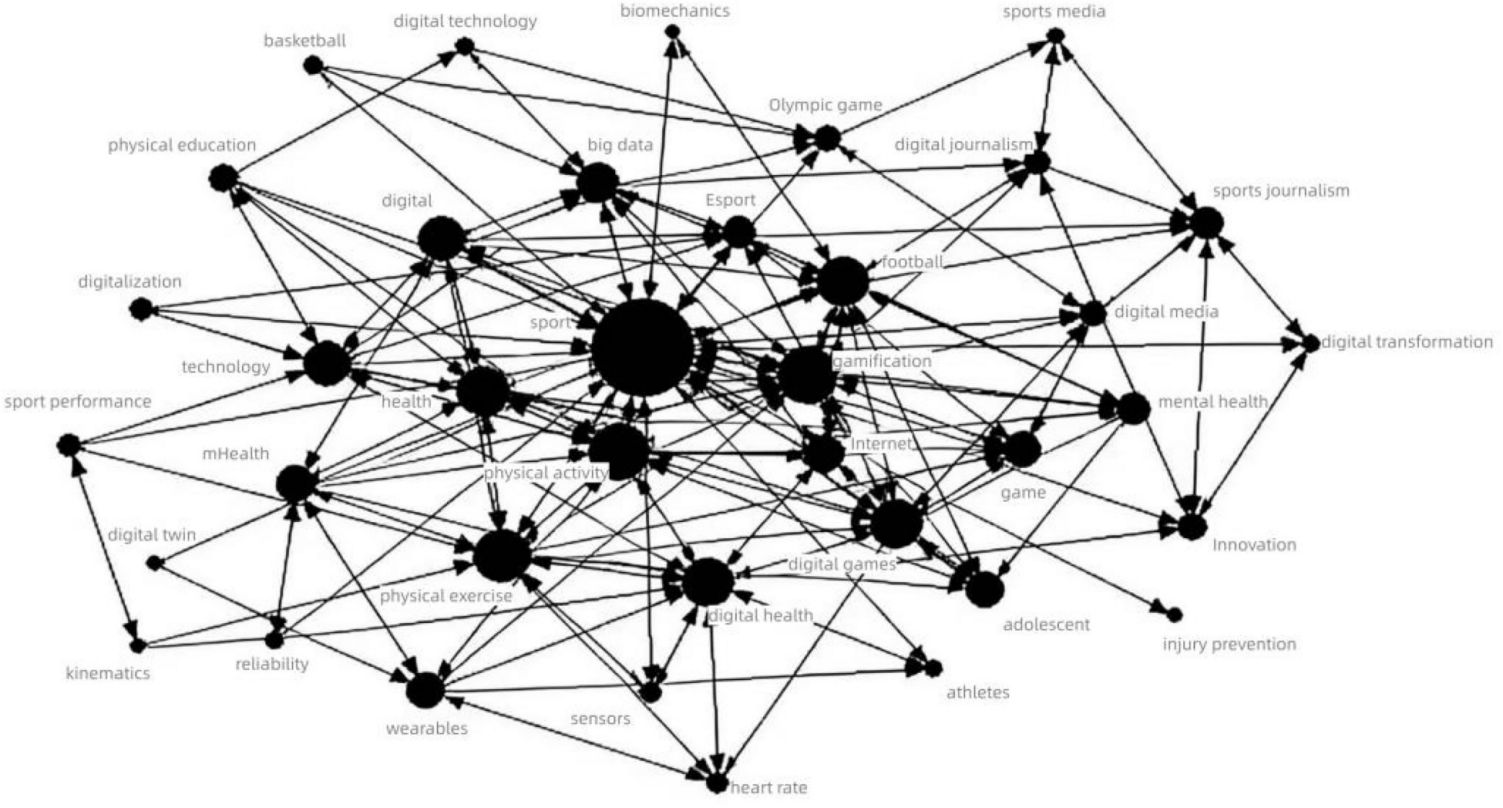
Social network graph of high-frequency keywords in digital sports. Node size represents the centrality of keywords (larger nodes indicate stronger control within the network), while edge thickness reflects the co-occurrence strength between keywords (thicker edges signify closer associations). The network exhibits a distinct “core-periphery” structure: the central area is dominated by keywords such as “exercise,” “physical activity,” and “gamification,” constituting the mainstream discourse of digital sports research. Peripheral nodes (e.g., “kinesiology,” “biomechanics”) are sparsely connected, suggesting limited dialogue with mainstream research.

To present the node centrality more intuitively and clearly, the co-occurrence matrix was imported into Ucinet 6.0 software, and the Netdraw drawing tool was used to create a social network graph of high-frequency keywords in digital sports (Figure 2). In the graph, circles represent high-frequency keywords; the size of the node represents its strength in controlling other nodes, and the thinner the connecting line, the closer the relationship between keywords. Judging from the size of the circular nodes, the main research focus in the field of international digital sports revolves around keywords such as “sport,” “physical activity,” “gamification,” “football,” “physical exercise,” “Digital games,” “Digital health,” “technology,” “digital,” and “Big data,” which also represent research hotspots in international digital sports research. From the perspective of relationships between nodes, “sport,” “gamification,” and “physical activity” are most closely connected. These three keywords revolve around the development direction of digital sports, indicating that current international attention is most focused on how digital technology affects sports. Next, “football,” “physical exercise,” “Digital health,” “Digital games,” “digital,” “technology,” “health,” “Internet,” and “adolescent” are relatively closely connected, indicating that the main areas of international concern are adolescents, focusing on how digital sports can promote healthy lifestyles, improve sports experiences, and prevent health issues. This involves using digital technology and big data to promote healthy exercise and football among adolescents, as well as how to use digital health methods to prevent and treat adolescent health problems.

It is noteworthy that, from the perspective of the overall social network, terms such as Kinesiology, Injury Prevention, Range of Motion, and Biomechanics are situated at the periphery. Their distribution is relatively scattered and independent, showing weak connections with other keywords. This phenomenon may reflect issues at several levels. First, it suggests the existence of knowledge barriers between disciplines. Although digital technologies have been widely applied in sports biomechanics research (e.g., motion capture, mechanical modeling), such studies are often published in specialized engineering or biomechanics journals. This creates a certain disconnect from the dominant discourse system of “digital sports,” which is largely centered around topics like “sports media” and “e-sports.” Second, this could stem from the fragmentation of research methods. Biomechanics research frequently employs experimental designs, whereas “digital sports” research tends to favor social science paradigms. The keywords used and the research traditions of these two areas have not yet fully converged. Nevertheless, this marginalization precisely points to future research opportunities. With the deep integration of wearable devices, artificial intelligence, and sports science, biomechanical and kinesiological data will become core supports for personalized training and injury prevention. Future research could explore how to better integrate these “hard science” findings into the sociocultural analytical frameworks of digital sports, thereby fostering interdisciplinary dialogue.

### 3.2 Co-word analysis

**Figure 3:**
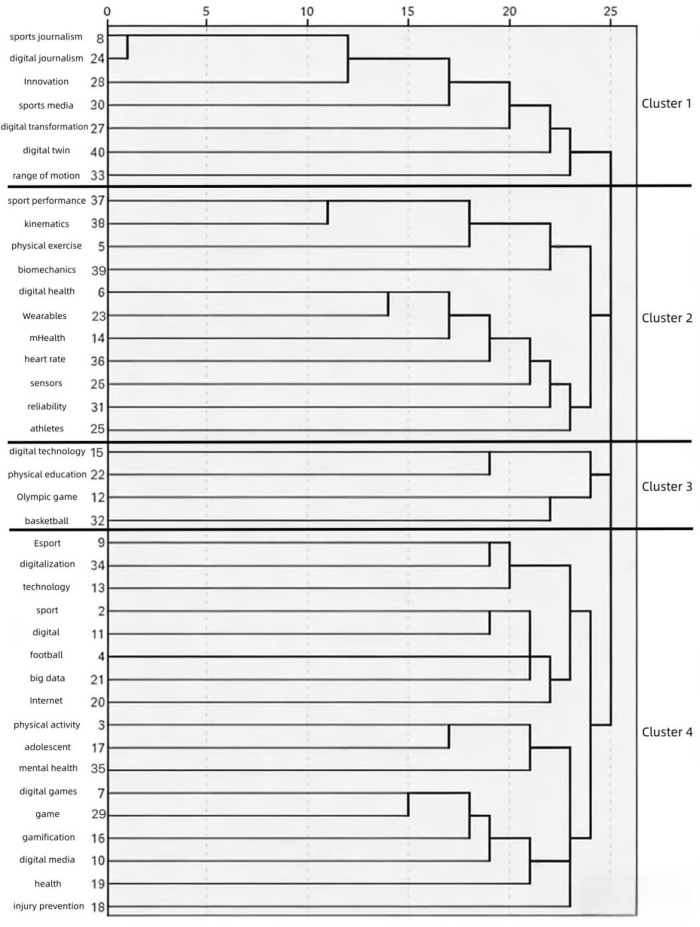
Cluster dendrogram of high-frequency keywords. The clustering method using between-groups linkage was employed, with the Ochiai coefficient serving as the distance measure. Keywords on the vertical axis are grouped into four clusters based on association strength, corresponding to Sports Media (Cluster 1), Sports Training (Cluster 2), Physical Education (Cluster 3), and Exercise & Health (Cluster 4). The clustering results reveal the four major thematic areas of digital sports research and the composition of keywords within each.

Cluster analysis groups closely related keywords together to form clusters. Using SPSS 26.0 statistical software, systematic cluster analysis was performed based on the similarity matrix of high-frequency keywords, resulting in a cluster dendrogram, as shown in Figure 3.

As can be seen from Figure 3, international digital sports research points are relatively concentrated and can be divided into four areas: Area 1 includes sports journalism, digital journalism, innovation, sports media, Digital transformation, digital twin, range of motion; Area 2 includes sport performance, kinematics, physical exercise, Biomechanics, Digital health, Wearables, mHealth, heart rate, sensors, reliability, athletes; Area 3 includes Digital technology, physical education, Olympic game, basketball; Area 4 includes Esport, digitalization, technology, sport, digital, football, Big data, Internet, physical activity, adolescent, mental health, Digital games, game, gamification, Digital media, health.

Through keyword semantic analysis and the co-occurrence matrix, each area is named respectively: Cluster 1, Digital Innovation Promoting the Development of Sports Media; Cluster 2, Implementation of Digital Devices in Sports Training; Cluster 3, Integration of Digital Technology in Physical Education; Cluster 4, Impact of Digital Transformation on Sports Health.

#### 3.2.1 Cluster 1: digital innovation promoting the development of sports media

This theme includes high-frequency keywords such as sports journalism, digital journalism, innovation, sports media, and Digital transformation. Sherwood et al. (8) examined the impact of digital social platforms on sports media, finding that digital platforms, as communication channels, can more effectively convey sports information, improve content quality, and are popular with the public. Meanwhile, digital technology is subtly changing the development of the sports media industry. Oelrichs (9) analyzed the use of Twitter by German sports journalists, finding that the platform enables live and on-demand services for sports events and, through data analysis, provides users with precise services and personalized content. This indicates that the rapid development of digital platforms is changing the symbiotic relationship between sports and media.

To explore the development of digital innovation in sports media, foreign scholars such as Hutchins et al. (10) analyzed from perspectives of social media anthropology, social psychology, and economics, finding that digital platforms, applications, and wearable devices play a dominant role in sports media culture in the information age. Digitalization brings users opportunities for social and human-machine interaction, promoting communication and sharing among users. To explore the challenges faced by sports media in the era of digital technology, scholar Ncube et al. (11), through in-depth semi-structured interviews with 15 sports journalists, found that a significant challenge for multimedia sports journalists is the limited application of digital devices in sports media. Therefore, there is an urgent need to explore and develop new digital devices for application in the sports media industry to promote the development of digital sports media. The addition of digital technology is driving traditional sports media into the era of digital sports media. Moritz (12), from a media sociology perspective, examined contemporary sports media through in-depth interviews with 25 sports journalists, finding that traditional sports media is highly institutionalized, but digital sports media is far less institutionalized.

During COVID-19, all industries were impacted, but it also presented a business opportunity to promote the development of digital sports media. German scholars (13), based on mediatization theory, through in-depth interviews with 32 German sports club personnel, found that digital sports media expanded opportunities for communication and sharing. The rapid development of esports, sports games, e-commerce, etc., brought new business opportunities to sports media. The rise of digitalization led to a significant increase in attention to digital sports media. Lee et al. (14), based on LDA topic modeling analysis of sports research related to COVID-19, identified four main themes: sports participation, sports performance, sports events (including professional leagues), and the sports industry.

It can be seen that international research scholars mostly use qualitative methods to analyze the development status of contemporary sports media. In the era of the metaverse, introducing new technologies and innovative methods enriches the content of sports media, while digital platforms, as frontier platforms, provide new profit models for digital sports media. Digital innovation brings new development opportunities and prospects to sports media, enabling digital sports media to gain favorable competitive advantages in the global metaverse market. The research progress within this cluster essentially represents a profound evolution of “sports communication” theory within the context of the platform society. It moves beyond traditional media effects research, shifting focus to how “platformization,” as a new socio-technical logic, restructures power dynamics, labor practices, and audience participation in sports communication (15).

#### 3.2.2 Cluster 2: implementation of digital devices in sports training

This theme includes high-frequency keywords such as kinematics, physical exercise, sensors, Wearables, and athletes. Physical health plays an important role in people's daily lives. Through physical exercise, life can be better enjoyed. At the same time, scientifically monitoring sports training and personal physical condition is very important. Due to increasing demand for virtual reality (VR) and augmented reality (AR), wearable systems that can measure human movement are being actively researched by many foreign scholars.

Sensors that perceive hand joint movement can assist in athlete training. Accurate measurement of human movement is crucial for interaction with virtual environments, especially the measurement of finger movement. Park et al. (16) proposed a three-dimensional (3-D) finger movement measurement system based on soft sensors, modeling complex finger joints [e.g., the carpometacarpal (CMC) joint of the thumb] to specify sensor positions, thereby achieving human-computer interaction. Wen et al. (17) detailed a user-friendly coating method using carbon nanotubes and thermoplastic elastomer (CNTs/TPE). The minimally designed glove has the potential to achieve complex gesture recognition, possessing strong energy harvesting and human motion sensing capabilities, performing various gesture recognition tasks in real-time through gestures to achieve high-precision virtual reality/augmented reality (VR/AR) control. To improve accuracy and usability, Connolly et al. (18) studied and described a unique method using neural networks for calibration and angle calculation. This method improves the repeatability and measurement accuracy of data gloves without calibration and is also suitable for athletes with limited joint mobility. They can also be made into human-computer interaction devices, providing athletes with scientific training methods.

Wearable sensors can provide assistance to athletes. A common issue with digital devices is: Are the obtained data accurate? Of course, this problem varies by application and usually only becomes known when the device is widely used in the market. Chen et al. (19) measured wrist-worn pedometers (e.g., in smartwatches) with errors between 1.5% and 9.6%, which have been successfully used to increase motivation to participate in physical activity (20) and help obese people lose weight (*p* < 0.001). Pedometers can provide users with quantitative feedback on physical activity levels (21). Han et al. (22) designed a self-powered wristband that can quantitatively detect walking steps, speed, and distance, and can also be used for identity recognition through gait monitoring and analysis (e.g., personal computer login and employee clock-in). This design inspires new ideas for human-computer interfaces.

Foot motion monitoring is crucial for sports training. Elstub et al. (23), by combining pressure-sensing insoles and shoe-mounted IMUs with algorithms, tested a peak tibial force with an average error of 5.7% during running. A wearable detection system monitoring tibial force can help understand and reduce injury incidence. Dragulinescu et al. (24) introduced smart socks for gait and foot pressure analysis, as well as the application of the Pedar-X system in sports, including running, basketball, badminton, and football, finding that both smart socks and the Pedar-X system can be used to guide athlete training and prevent foot injuries.

It can be seen that international research scholars pay more attention to the development of digital devices in sports and focus more on the development and application of smart devices. Integrating smart electronic devices into sports training can make athlete training more scientific and convenient. Therefore, the next step should focus on developing real-world wearable devices and emphasize longitudinal studies to apply them in sports training. This cluster highlights the paradigm shift in sports training science from experience-based guidance to “data-driven” decision-making. This echoes the socio-technical discourse of “Quantified Self” and the “datafication of the body,” wherein the athlete's organism is transformed into a real-time monitored, analyzed, and optimized data stream (25). The latest research trends indicate that artificial intelligence is advancing from basic data processing toward more complex prediction and decision support. For instance, in team sports contexts, proof-of-concept models based on machine learning have been applied to the selection and performance prediction of elite athletes (26), signaling the evolution of data-driven decision-making toward higher-order intelligent assistance systems.

#### 3.2.3 Cluster 3: integration of digital technology in physical education

This theme includes high-frequency keywords such as Digital technology, physical education, Olympic game, and basketball. With the rapid development of network and computer technology, the speed of knowledge updating is accelerating day by day, and educational methods are gradually changing. The traditional “teacher teaches, student learns” teaching method can no longer meet the needs of contemporary students. The integrated development of digital technology and physical education has become a new trend.

Fu (27), through questionnaire surveys, found that students believe digital teaching has a higher impact on physical education teaching than traditional teaching. Digital teaching can increase students’ learning interest more than traditional teaching models, strengthen mutual trust and cooperation between teachers and students, and improve students’ practical abilities. Traditional physical education teaching methods are facing increasingly severe challenges. Xue et al. (28) investigated and analyzed the current situation of online teaching platforms and course resources in 12 higher physical education institutions, proposing a management platform model that organically combines online teaching platforms with digital teaching resource databases. Through the application of this technology, the quality of online physical education can be improved, teaching costs reduced, and students provided with a higher-quality and more efficient learning experience.

Educational informatization is a key area of current educational reform. Conducting online physical education courses has become a new trend, and digital information platforms are new ways to conduct online physical education teaching. Wu et al. (29), based on the auxiliary analysis of a digital platform using IoT technology in physical education teaching, proposed a radio frequency algorithm with high recognition rate in the process of sports gesture recognition, which can accurately assist the conduct and completion of physical education teaching. This platform makes online teaching in physical education possible, provides new methods for physical education teaching, enriches teaching resources, improves teaching efficiency, and promotes the improvement of physical education teaching quality. To analyze more deeply the opportunities and challenges brought by digital technology in physical education, Ruin et al. (30) found that using digital technology, students can experience the difference between objective and subjective perspectives of the body and movement behavior, constituting, in a sense, a form of cultivation-oriented digital health education.

Digital technology transformation has greatly changed the way contemporary sports events are produced and consumed worldwide. Ludvigsen et al. (31), through a case study using the video-sharing platform YouTube as an example of the Olympic Games’ digital transformation, help other scholars strengthen their understanding of physical education, major sports events, and digital platforms. Faced with educational informatization, physical education teaching methods and means still remain in traditional verbal and physical teaching. To explore new paths, Ding et al. (32), based on IoT wireless channel technology and UWB indoor positioning technology, designed a physical education course scheduling system and a student positioning and check-in system, establishing a personalized physical education teaching system. This helps solve problems such as shortage of physical education teachers and conflicts between learning and training, and can save costs and improve resource utilization efficiency.

In summary, integrating digital technology into physical education has become a catalyst for reshaping learning experiences, promoting innovation, and preparing individuals for the digital age. Making full use of digital technology in physical education teaching can optimize teaching elements, enrich teaching resources, restructure teaching models, and promote the transformation of physical education towards digital informatization teaching. This cluster reflects the intersection of educational technology theory and sports pedagogy. Its core theoretical issue lies in how “technological mediation” reshapes bodily experience, teaching interactions, and learning outcomes (33). While research indicates that digital tools can enhance motivation and personalized learning, a critical perspective must question: To what extent can virtual experiences replace or enhance the unique bodily perception, social interaction, and risk-based learning inherent in physical education? Furthermore, unequal access to technology may exacerbate the “digital divide” in physical education, which is an important sociological issue concerning educational equity.

#### 3.2.4 Cluster 4: impact of digital transformation on sports health

This theme includes high-frequency keywords such as Esport, digitalization, technology, sport, mental health, Digital media, and health. With the continuous rapid development of technology, digitalization has permeated all aspects of sports and athletic performance. The impact of digital transformation on sports health is multifaceted, providing new opportunities and challenges for athletes, coaches, and sports psychologists.

To build a health network intelligent management system, Xia et al. (34) started from infusion monitoring in clinical medical care. The sports rehabilitation training system receives EEG signal data collected by the UE-16B EEG signal amplifier through a socket, calls MATLAB for feature extraction and classification of EEG signal data, and feeds the processing results back to the sports rehabilitation training system and the subject. Additionally, the system designed and completed non-feedback training modules, feedback training modules, and related BCI game modules, promoting users to perform motor imagery training and successfully complete sports rehabilitation training. The human body is a complex system, and accurate prediction of exercise fatigue level has certain challenges. Yao (35) used machine learning algorithms to analyze and process data collected by sensors, learning data patterns and making predictions based on these patterns. This method can provide coaches and athletes with timely and accurate fatigue monitoring, thereby helping to optimize training plans and avoid sports injuries. Addressing the serious problem of knee injuries among elite track and field athletes, Wang et al. (45), based on biomechanics, used surface electromyography (iEMG—Integral EMG value) to guide, amplify, display, and record changes in bioelectricity during local neuromuscular system activity to prevent athlete knee injuries, aiming to provide coaches with a basis for improving technical levels and training.

As digital media becomes increasingly important for young athletes, research exploring athletes’ mental health is equally important. Fiedler et al. (36) examined 591 German youth athletes (12–19 years old) from 42 different sports. It was found that the longer the daily use of social media, the more negative emotions increased, and eating patterns would become disordered. Similar results were found in the relationship between excessive media use and cognitive-behavioral symptoms of mental health. The negative correlation between excessive media use and sleep was stronger in competitive and elite athletes than in recreational athletes. Mental health cannot be separated from physical health. Mental health symptoms and disorders increase the risk of physical injury and delay subsequent recovery. Reardon et al. (37) emphasized the need for a more standardized, evidence-based approach to mental health symptoms and disorders in elite athletes. To explore the dynamic intersection of sports psychology and emerging technologies, Li et al. (38), through literature retrieval and keyword trend analysis, found that the impact of social media on athletes’ mental health cannot be ignored, as it affects self-esteem, body image, and overall mental well-being. Therefore, it is necessary to understand the impact of social media on athletes’ mental health and provide targeted support and interventions.

Flexible wearable sensors have high stretchability and portability and can be widely used in health diagnosis, sports monitoring, rehabilitation, and other fields. Peng et al. (39) prepared a photocurable resin containing ionic liquids (ILs) and hydrogen bond-rich acrylate monomers. Photocurable resin is a promising material for digital light processing (DLP) 3D printers, which can print customized PIFS with high stretchability for monitoring pulse, fingers, gait, and other human movements. Currently, wearable electronics for gait analysis are mainly limited by high manufacturing costs, high operational energy consumption, or inferior analysis methods. Zhang et al. (40) developed low-cost triboelectric dielectric smart socks, establishing a digital human system for sports monitoring, sports health, identity recognition, and future smart home applications.

It can be seen that international research scholars mostly approach from the perspectives of medicine, biomechanics, sports training, etc., exploring the impact of digitalization on sports health in multiple dimensions. The application of intelligent digital technology (such as smart wearable technology) has evolved from collecting and analyzing biological information to monitoring environmental changes and interacting with users. Digital devices provide new possibilities for monitoring and analyzing athlete performance, thereby driving progress in the field of sports science. It can be concluded that analysis and statistical tools, wearable devices, and management information systems are tools that have a significant impact on the sports industry. This cluster most prominently illustrates the “health paradox” of digital sports, providing rich case studies for the “sociology of digital health” (41). This calls for researchers to move beyond simplistic narratives of technological efficacy and adopt a dialectical “socio-technical systems” perspective to examine how technological design, business models, individual usage, and socio-cultural environments collectively shape the health outcomes of digital sports.

## 4 Discussion

The knowledge map drawn in this study indicates that digital sports is a cross-disciplinary field constructed through multiple sociotechnical logics. The following sections will provide an in-depth interpretation of these four thematic clusters by integrating relevant theoretical perspectives, in order to reveal the underlying academic debates and theoretical implications.

### 4.1 “Platformization” of sports media and power restructuring

The research findings of this cluster are highly consistent with the “platform society” theory proposed by Van Dijck et al. (15). This theory posits that digital platforms have transcended their instrumental attributes to become infrastructures that reshape social institutions, economic relations, and power dynamics. Digital platforms have not only transformed content distribution channels but have also reshaped the power dynamics and value flows among producers (journalists, clubs), consumers (fans), and platform corporations, giving rise to new forms of participatory culture and digital labor (10). The shift in research focus from traditional institutional media to social media precisely mirrors this process of power restructuring in academic attention, suggesting that the power of sports communication is undergoing a process of dispersion and recentralization, with platform corporations emerging as new key actors (14). This implies that sports communication research urgently needs to evolve from traditional media effects analysis toward a critical examination of issues such as platform political economy, algorithmic governance, and digital labor. This transformation requires an upgrade of the traditional sports information management paradigm to advance the digitalization paradigm of sports. This evolution indicates that digital sports media are no longer merely an extension of traditional sports but have formed a new value network centered on platforms. Its core characteristics include the decentralization of content production, the prosumerization of audiences, and data-driven precision communication. Compared to previous reviews that focused solely on the application of media technologies, this study reveals how “platformization,” as an underlying logic, reconstructs the ecological structure of sports communication and raises concerns about deeper issues such as platform governance, algorithmic transparency, and digital labor rights. Future research needs to further explore the impact of platform monopolies on the diversity of sports culture, as well as the local manifestations of platformization in non-Western contexts.

### 4.2 “Datafication” of the body and changes in training science paradigm

The research trends within this cluster resonate with the “quantified self” theory advanced by Lupton (42), which suggests that wearable devices and sensors transform athletes’ bodies into data streams for continuous monitoring, analysis, and optimization. This drives the shift in training management from experience-based judgment to a data-driven paradigm. Wearable devices and sensors transform athletes’ bodies into continuous data streams. This not only drives a paradigm shift in training management from empiricism to evidence-based science but also situates sports science within broader social theoretical discussions such as the “quantified self” and “biological citizenship.” The ensuing debates over data ownership, athletes’ privacy rights, and the challenge of technological reliance to coaches’ experiential judgment have become central issues in sports ethics. Training science in the context of digital sports is undergoing a paradigm shift from “experience-led” to “data-driven.” This shift involves not only technological application but also a transformation in the epistemology of trainingorts is unde's body is reconfigured as a quantifiable and predictable “data body.” Compared to previous reviews that emphasized technological efficacy, this study reveals the reconstruction of the human-technology relationship behind datafication: the coach's role evolves from an experience imparter to a data analysis collaborator, while athletes face the risk of being disciplined by data. The key in the future lies not in the infinite stacking of technology, but in exploring a training philosophy of “data-experience” synergy and establishing mechanisms to protect athletes’ data sovereignty. This requires interdisciplinary research integrating sports science, data ethics, and artificial intelligence, rather than simplistic suggestions for talent cultivation.

### 4.3 Technology-mediated and experience reshaping in educational contexts

The research findings of this cluster align with the theoretical framework of “technology-mediated learning” proposed by Casey et al. (43). Digital technologies, acting as mediators, can reshape pedagogical interactions, learning experiences, and knowledge transmission methods within physical education. Cluster 3 indicates that digital technology is being actively introduced to address traditional challenges in physical education, such as personalized teaching, resource shortages, and increasing participation motivation (27, 29). However, a core theoretical and practical question remains: Can virtual experiences substitute for real physical interaction and social learning, and to what extent? Does technology deepen or bridge educational inequality? To resolve these tensions, it is necessary to move beyond an application-oriented mindset rooted in instrumental rationality and promote the critical integration of digital technologies in physical education. This is, in essence, an exploration of how to achieve a creative balance between a philosophy of “technology-enabled empowerment” and one of “body-centered” teaching in sports education. It can thus be seen that the evolution of digital sports education is not a simple accumulation of technology, but rather raises fundamental pedagogical questions—how is “embodied learning” possible through technological mediation? Previous reviews have largely affirmed the positive effects of technology. This study, however, reveals the inherent tensions brought by technological embedding: the dialectical relationships between efficiency and experience, the virtual and the real, and inclusivity and the digital divide. Future research needs to shift from “technology application” to “pedagogical reconstruction,” exploring design principles for bodily perception and social interaction in blended learning environments, and paying attention to how technology affects the equity of learning opportunities for different student groups. This perspective elevates physical education research from instrumental rationality to the level of educational philosophy.

### 4.4 Health contradictions of digital participation and social inclusivity

This cluster reflects the “sociotechnical systems” theory articulated by Bostrom & Heinen (44), which posits a complex interaction among technology, individuals, and the social environment in shaping the relationship between digital technologies and health outcomes. Cluster 4 is the largest, highlighting the complexity of the social impact of digital sports. On the one hand, “digital health” and “gamification” point to the potential of technology to promote active lifestyles; on the other hand, the co-occurrence of keywords such as “esports,” “mental health,” and “adolescent” reflects academic concerns about risks such as excessive screen time, cyberbullying, and occupational health (37). This reveals a core paradox of digital sports: while it expands social participation (especially for certain marginalized groups), it may also create new forms of exclusion and health risks. Compared to previous reviews that often focused on unidimensional assessments of technological efficacy or risk, this study, through knowledge mapping, reveals the coexistence and tension between these two dimensions within the same field. It indicates that digital sports health research is shifting from a “tool effectiveness” paradigm to a “sociotechnical systems” paradigm. This necessitates a comprehensive examination of how technological design, commercial motives, individual behavior, and sociocultural environments jointly construct health outcomes. Future research must go beyond validating the effects of specific technologies, delve into the differentiated risks and benefits of digital participation among diverse groups (e.g., professional athletes, adolescents, the elderly), and develop health intervention models with greater ecological validity.

## 5 Conclusion

Through co-word analysis, this study systematically outlines the macro landscape of international digital sports research over the past two decades. Based on the analysis of the four clusters above, this study has developed the following understanding of the evolution of the digital sports field: (1) Confirmed research consensus includes: Digital technologies have extensively permeated sports media, training, education, and health, forming four relatively stable thematic clusters. Core research hotspots such as “exercise,” “gamification,” and “physical activity” have been established. (2) The novel findings of this study are mainly reflected in: For the first time, it systematically reveals the disconnect between the four clusters—the marginalization of traditional “hard sciences” like biomechanics and kinesiology within digital sports research, indicating persistent barriers to interdisciplinary integration. It also identifies deep sociotechnical logicsystematic“platformization,” the “datafied body,” and the “health paradox”—ealth paradoxn,stent barriers to interdisciplina perspectives for understanding the complexity of digital sports. (3) Research directions that have not yet been fully explored include: Non-Western experiences and localized practices in digital sports, pathways for integrating biomechanics with social science research, and the long-term impacts of digital technologies on equity in sports participation and ethical risks. These unresolved questions provide clear directions for future research.

Theoretical Implications: The knowledge map provided by this study offers a “navigation map” for subsequent theoretical research. Scholars can further explore on this basis: (1) How digital sports reconstruct power relationships in the sports field (e.g., among platforms, institutions, and individuals); (2) The impact of bodily datafication practices on sports body culture and athlete subjectivity; (3) The globalization process of digital sports and its adaptive tensions in different local contexts.

Research Limitations and Future Directions: This study is based only on English journal literature; future research could include other languages and grey literature to obtain a more comprehensive view. In addition, dynamic evolution analysis could more precisely capture the temporal changes in research hotspots. Finally, it should be noted that the findings of this study, based on co-word analysis, are subject to limitations including the quality of keyword indexing, database selection bias, and the inherent constraints of the method itself in inferring causality. These factors constitute the limitations of this research.

## Data Availability

The original contributions presented in the study are included in the article/Supplementary Material, further inquiries can be directed to the corresponding author.
